# PSO-SVM-Based Online Locomotion Mode Identification for Rehabilitation Robotic Exoskeletons

**DOI:** 10.3390/s16091408

**Published:** 2016-09-02

**Authors:** Yi Long, Zhi-Jiang Du, Wei-Dong Wang, Guang-Yu Zhao, Guo-Qiang Xu, Long He, Xi-Wang Mao, Wei Dong

**Affiliations:** 1 State Key Laboratory of Robotics and System, Harbin Institute of Technology (HIT), Harbin 150001, China; scdxhgd@gmail.com (Y.L.); duzj01@hit.edu.cn (Z.-J.D.); wangweidong@hit.edu.cn (W.-D.W.); 2 Weapon Equipment Research Institute, China Ordnance Industries Group, Beijing 102202, China; yuzmoon@126.com (G.-Y.Z.); xuguoqiang1988911@163.com (G.-Q.X.); helong208@126.com (L.H.); mayu-8714@163.com (X.-W.M.)

**Keywords:** SVM, PSO, locomotion mode identification, feature extraction, MVA, rehabilitation exoskeleton

## Abstract

Locomotion mode identification is essential for the control of a robotic rehabilitation exoskeletons. This paper proposes an online support vector machine (SVM) optimized by particle swarm optimization (PSO) to identify different locomotion modes to realize a smooth and automatic locomotion transition. A PSO algorithm is used to obtain the optimal parameters of SVM for a better overall performance. Signals measured by the foot pressure sensors integrated in the insoles of wearable shoes and the MEMS-based attitude and heading reference systems (AHRS) attached on the shoes and shanks of leg segments are fused together as the input information of SVM. Based on the chosen window whose size is 200 ms (with sampling frequency of 40 Hz), a three-layer wavelet packet analysis (WPA) is used for feature extraction, after which, the kernel principal component analysis (kPCA) is utilized to reduce the dimension of the feature set to reduce computation cost of the SVM. Since the signals are from two types of different sensors, the normalization is conducted to scale the input into the interval of [0, 1]. Five-fold cross validation is adapted to train the classifier, which prevents the classifier over-fitting. Based on the SVM model obtained offline in MATLAB, an online SVM algorithm is constructed for locomotion mode identification. Experiments are performed for different locomotion modes and experimental results show the effectiveness of the proposed algorithm with an accuracy of 96.00% ± 2.45%. To improve its accuracy, majority vote algorithm (MVA) is used for post-processing, with which the identification accuracy is better than 98.35% ± 1.65%. The proposed algorithm can be extended and employed in the field of robotic rehabilitation and assistance.

## 1. Introduction

An automatic and smooth locomotion transition is important for many kinds of robots such as bipedal robots, lower extremity exoskeleton robots and prostheses [1,2,3]. In some real situations, it is necessary to know about the locomotion mode condition and preceding corresponding movements to complete tasks. In addition, an adaptive smooth locomotion transition can enhance safety and balance to bring about a more natural gait pattern. To achieve a smooth transition between different locomotion modes such as stair descent, stair ascent, ramp descent, ramp ascent, and level-ground walking, increasing efforts have been made during the last few decades. Approaches for locomotion mode identification can be classified according to the type of identification signals: classifiers using biological signals, e.g., EMG and other classifiers using kinesiological signals, e.g., interaction forces. The former category is dependent upon the EMG signals. In the work of Joshi et al., EMG data is collected from seven leg muscles while the subjects walk on the floor and ascend as well as the transition between them. Through the time-frequency representation of collected EMG data, simple if-else 81 rules with prior knowledge are applied to classify those two gait patterns and the transition [4]. Based on the collected EMG signals, six time-domain features for correlation feature analysis are used to identify different locomotion patterns, whose accuracy and stability are better than that using the method of principal component analysis (PCA) and linear discriminate analysis (LDA) [5]. An approach was proposed to detect the pattern of stair descent based on EMG to achieve the transition from level-ground walking to stair walking for the amputee’s residual limb [6]. Although the EMG signal is produced prior to motion, it is not sufficient to classify several locomotion modes independently [7]. The fusion of EMG signals and ground reaction forces/moments is used as the input of a phase-dependent pattern classifier using SVM, with which the locomotion mode identification accuracy can reach 99% in the stance phase and 95% in the swing phase [8].Young et al. proposed to combine EMG signals and signals from mechanical sensors such as IMU and load cells together, based on which a dynamics Bayesian network was used to identify basic locomotion modes such as walking, slopes and stairs to minimize the transitional error and steady state error [9]. Joshi et al. proposed to collect data with accelerometers and EMG electrodes on the dominant leg, with which LDA and SVM were used to classify normal gait patterns and the transitions between them [10]. Since EMG electrodes have to be firmly attached to the skin, it is not convenient for applications in real situations [11]. 

The other category is based on kinesiological signals containing kinematics information such as inclination angles and dynamics information such as force and moment signals. Ground reaction force (GRF) is collected using a wearable plantar pressure measurement system, based on which, LDA was used to achieve a good identification accuracy for several locomotion modes [12]. Although GRF can be collected easily, it only represents the interaction information between the robot and the ground, which will be invalid when the leg is located in the swing phase or on uneven surfaces [13]. The IMU can also be used to identify several gait patterns such as level ground walking, stair walking, and ramp walking with an optimized threshold method, based on which, the controller is able to identify gait modes in real time and properly control the actuation [14]. Based on previous sensor information and current sensor information of load cells and 6-axis IMU, a dynamic Bayesian network was used to identify several different gait patterns and achieve the transitions between locomotion modes [15]. In general, signals from single kind of kinematics or dynamics sensors are not sufficient for locomotion mode identification. Multisensory information has been widely used in locomotion mode identification for robots, which can improve system performance as well as robustness [16]. Based on angular positions and velocities of robots joints and interaction forces/moments between the user and the robot, Gaussian mixture model was used to classify activity modes with features dimension reduction using LDA [17]. Two foot pressure insoles and three inertial measurement units were used to identify six kinds of locomotion modes, i.e., stair ascent, stair descent, ramp ascent, ramp descent, level-ground walking and standing, where a LDA classifier was used with an accuracy of 99.71% [18]. Yuan et al. pointed out that a good terrain identification method should meet four requirements, i.e., high identification accuracy, minimal sensors which should be embedded into the mechanism, short identification delay, and low computation load [19]. 

For classification problems, there are several widely used algorithms such as LDA, Bayesian network, SVM, boosting, C4.5 decision trees, random forests, and neural network. SVM models are based on the statistical learning theory to achieve structural risk minimization and are a set of related supervised learning methods used for classification and regression [20]. SVM models possess the well-known ability of approximating any multivariate function to any desired degree of accuracy. In order to obtain the optimal hyper-parameters setting in the SVM training, several optimization methods such as grid search [21], genetic algorithm [22], PSO [23] are used with success. The PSO technique is a population-based search algorithm based on the simulation of the bird flocking and exploits the model of social sharing of information [24], which has been used widely in the optimization of SVM. A hybrid PSO optimized SVM (PSO-SVM) model was used as automated learning tool, trained in order to predict other parameters such as optical density, dissolved oxygen concentration, pH, nitrate concentration and phosphate concentration [25].

In this paper, we propose to use the SVM algorithm optimized by PSO to identify five kinds of locomotion modes, i.e., level-ground walking, stair descent, stair ascent, ramp ascent, ramp descent, and transitions between them. The source signals are collected from foot pressure sensors placed in the wearable shoes and AHRSs attached on the mechanical leg segments. Since GRFs measured by pressure sensors are invalid during the swing phase, the proposed identification algorithm is dependent upon phase events detection. The collected signals are processed by the following steps, i.e., noise eliminating with low-pass filter, feature extraction using WPA, feature dimension reduction using kPCA and normalization into the interval of [0, 1]. During the training process of the SVM, five-fold cross validation is used to prevent over-fitting. The model produced by the SVM is trained offline in MATLAB, based on which, the online SVM algorithm can predict the locomotion mode according to the signals measured by sensors in real time. The majority vote algorithm is used to improve its identification accuracy. To verify the proposed algorithms, experiments on different terrains are performed. 

The remainder of this paper is organized as follows: the robotic exoskeleton system is presented and the signal processing is described in the second section. In the third section, the locomotion modes are identified with SVM optimized by PSO. Experiments using the proposed approach and results analysis are presented in the fourth section. Conclusions are drawn in the fifth and final section.

## 2. Data Collection and Processing

### 2.1. Exoskeleton System Description

**Figure 1:**
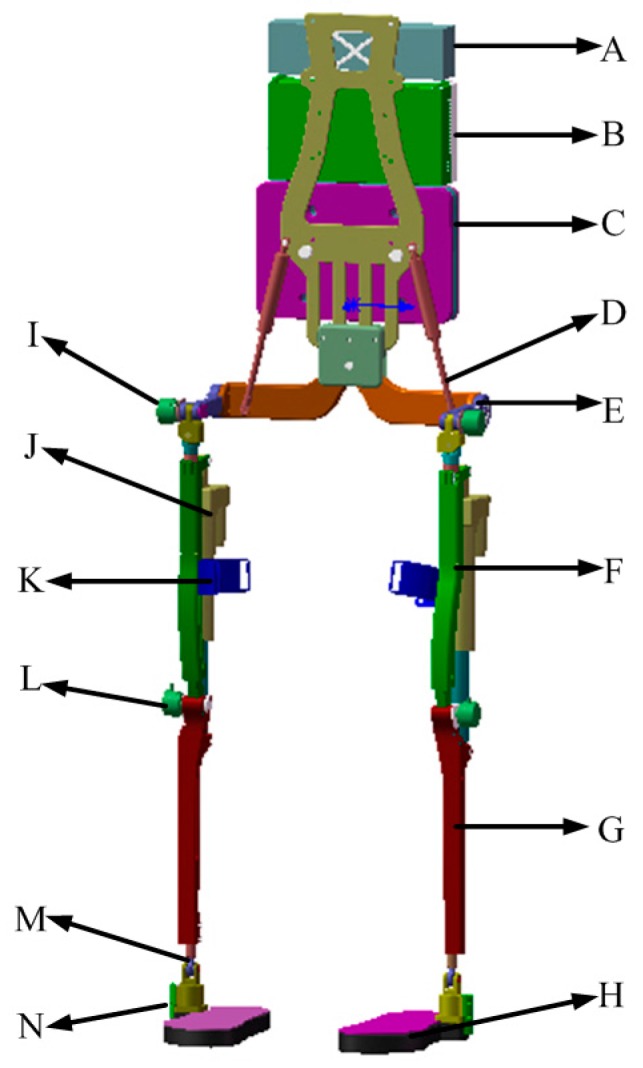
Mechanical structure of the exoskeleton. **A**: battery power source; **B**: control system enclosure; **C**: carried load; **D**: springs connecting the backpack with the waist part; **E**: waist part; **F**: thigh segment; **G**: shank segment; **H**: wearable shoes which have pressure sensors; **I**: optical encoder on the hip joint; **J**: actuation system consisting of DC motor, gear ratio and screw; **K**: connection cuff interacting with human limbs; **L**: optical encoder on knee joint; **M**: ankle joint; **N**: signal transfer for foot pressure.

The lower extremity exoskeleton has two seven degrees of freedom (DOF) mechanical links attached to human limbs, placed on the thigh, knee and ankle joints, respectively. Figure 1 shows the structure of the exoskeleton robot, with its three main components: (1) wearable shoes connected with the shanks, each of which contains six pressure sensors to collect ground reaction force information; (2) leg segments including shanks and thighs, which are attached to the human user’s legs by the interaction cuffs placed on the thigh segment of each leg. The knee joint is active while the hip joint is passive. The actuator of the knee joint consists of a DC actor and a screw, which helps legs walk and support the weight of system when the leg is located in the phase of stance; (3) trunk, which is used to package the control system, the power supplies and the loads to be carried. The trunk is connected with the leg segments on the back of the user. We take into consideration safety and comfort during the design of the whole exoskeleton system. To ensure safety in use, some mechanical limit braces, which are mounted on the movement joints, are utilized to insure that movement of joints is located in an appropriate zone. For comfort consideration, some elastic elements are utilized to resist impact. There are springs installed on the connection parts between the trunk and the leg segments. Besides, some soft materials are affixed into the mechanical braces of corresponding joints. Some mechanical parameters of the exoskeleton system are shown in Table 1. Table 1 describes the length and mass of thigh and shank of each leg. In addition, the range of movement of hip and knee joints is also presented. 

### 2.2. Data Collection System

**Figure 2:**
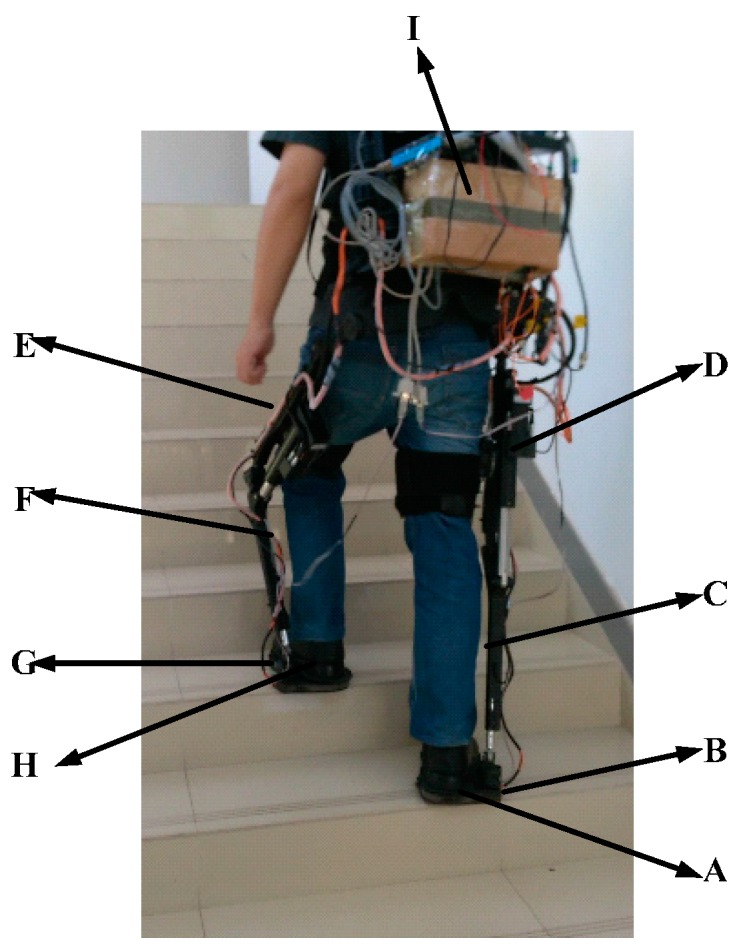
Mechanical structure of the robot used in this study. **A** and **H**: wearable shoes where three pressure sensors are placed in each; **B** and **G**: AHRS sensors which collect the inclination angles of foot for double legs; **C** and **F**: AHRS sensors which are attached to the leg segments to collect the inclination angles of shanks; **D** and **E** are motors with screws for the system; **I**: the central control system which collects data and produces control signals.

In different locomotion modes, the postures of the leg segment and the foot are the most important information. The movement in the sagittal plane contains the most valuable information for locomotion mode identification. In this paper, two kinds of sensors are used to collect the GRF information and the inclination angles of foot and shank. The GRF information is measured by an integrated insole with embedded force sensors. The GRF sensor can measure the weight up to 100 kg and has a resolution of 0.1 N. The inclination angles of the foot and the shank are measured by AHRS sensors. This kind of AHRS can measure the rotation angle directly with a resolution of 0.01°. Due to the existence of the measurement noises, the GRF information and the inclination angle are processed by a low-pass filter with a cut-off frequency of 50 Hz. The installation architecture of sensors is shown in Figure 2. 

**Figure 3:**
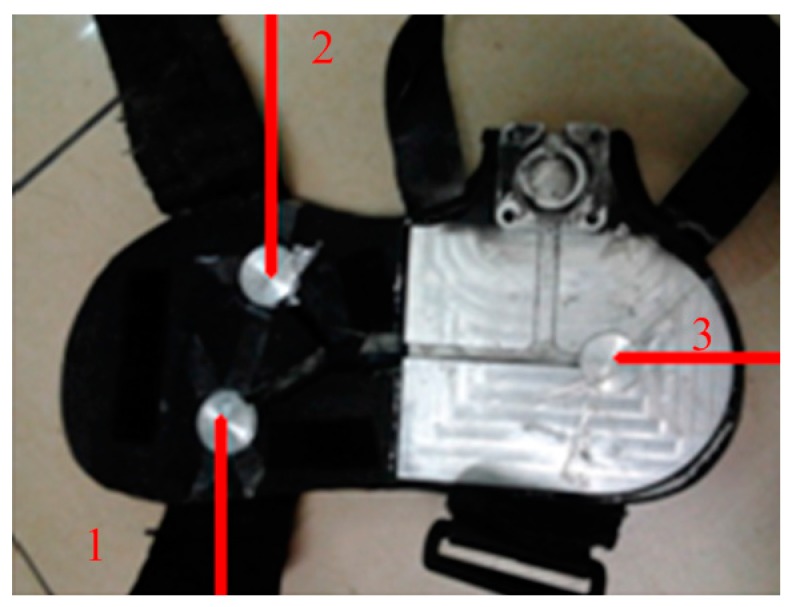
The placement of GRF sensors in the wearable shoe. Two GRF sensors are arranged on the forefoot and one sensor is placed on the heel. The labels **1**, **2** and **3** represent the three sensors.

As Figure 2 shows, GRF signals are collected by pressure sensors placed in the insole of the wearable shoes and the inclination angles of foot and shank are measured by AHRSs attached to the foot and the shank of legs. The GRF measurement insoles are self-made, based on the distribution measurement by the pressure insole produced by Tekscan (Boston, MA, USA) [26]. When the human walks on the floor, there are three zones under greater force distribution. The selected sensor can output the voltage signal proportional to the applied force. This kind of sensor has a high enough resolution to record the GRF changes during the stance period. In addition, this kind of sensor is small and thin enough to be embedded into the wearable shoes and to ensure the comfort. The proposed wearable shoe is shown in Figure 3. 

The GRF sensor has a sensing area of 4.52 cm^2^ and has a measurement ranging from 0 kg to 100 kg. MEMS-based AHRS sensors (AHRS II, SkyLark, HongKong, China) are used to collect the movement information of the leg segment, especially when the leg is in the swing phase, during which the GRF has extremely small outputs. The AHRS includes gyroscopes, accelerometers and magnetometers and is capable of outputting three-axis (Roll, Pitch and Yaw) rotation posture, speed, and acceleration. The rotation angle can be obtained through the integration of angle speed. The accuracy of measurement can be improved by the compensation of the information fusion algorithm, e.g., extended Kalman filter. In this kind of used AHRS, the extended Kalman filter is used to deal with the problem of time drift and output accurate data in real time. The AHRS can output the measurement angles directly with a measurement range of (−180°, 180°), (−180°, 180°) and (−90°, 90°) in the Roll angle, the Yaw angle and the Pitch angle, respectively. The output of the AHRS can be transferred to the host computer through a standard RS-232 connector. The sampling frequency is 40 Hz and the baud rate is set as 115,200. The Roll angle is used for the measurement of the human limb gait trajectory. The AHRS, which are fixed on the middle of the shank and the side of the foot, are applied to measure the inclination angle of the shank and the foot.

**Figure 4:**
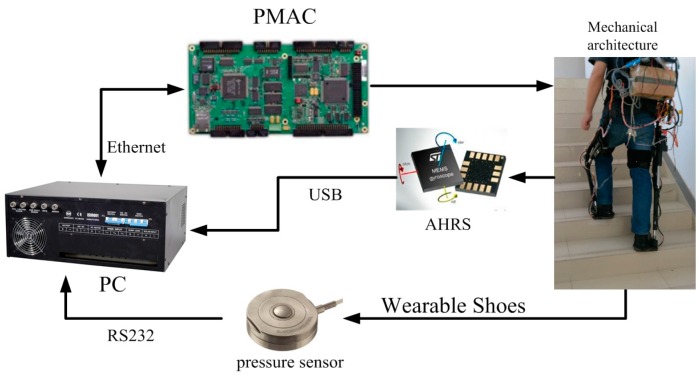
The signal collection structure. The measuring range of pressure sensors is 0~100 kg while that of AHRS sensors is −180°~180° in the sagittal plane.

**Figure 5:**
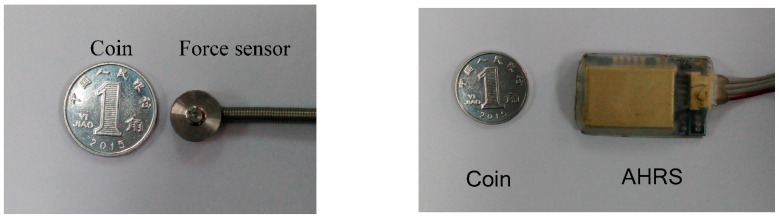
The GRF sensor and the AHRS sensor of the system, which are small and thin to be embedded in the mechanical structure.

During a single sampling interval, the information of the GRF sensors and the AHRS sensors is collected and transferred to the PC. The signals collection structure is described in Figure 4. AHRS sensors can collect the inclination angles in sagittal plane, which are sent to the PC through USB. Pressure sensors are placed in the insole of the wearable shoes to collect GRF during walking, which is transferred to the PC through a standard RS232 bus. A lithium battery (12 V, DC) is utilized to provide power for the system. Sampling rates of those sensors are 40Hz. Signals processing and identification algorithms run in the PC. The PC sends the identified locomotion mode into the Programmable Multi Axis Controller (PMAC, Delta, Atlanta, GA, USA), which conducts motion planning for the robot. The applied force sensors and AHRS sensors are depicted in Figure 5.

**Figure 6:**
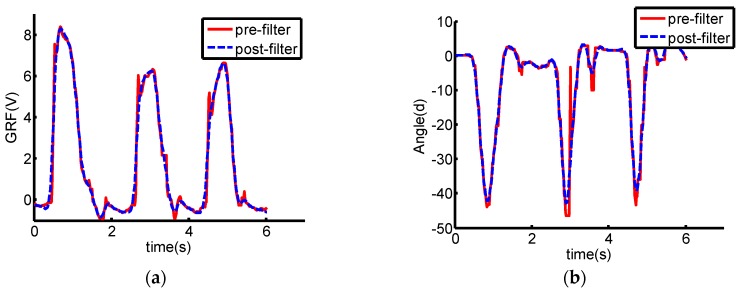
Signal curves comparison between pre-filter and post-filter. Signals through filter are smoother. (**a**) GRF curves from the sensor placed on the heel; (**b**) Inclination angle curves from the AHRS on the foot.

Three GRF sensors are placed on the insole to measure the GRF and the data can be collected by standard RS-232 bus. AHRS sensor signals can be directly collected through a USB connection. The collected GRF signals and the inclination angle are processed with the Kalman smoother, which has two main functions, i.e., (1) eliminate the high frequency noise signals as the general low-pass filter does and (2) predict the signals forward for a step and smooth the collected signals. The processing results are depicted in Figure 6. Compared with the original signals, the Kalman smoother is capable of smoothing the signals. Only the flexion and extension DOF of the knee joint is actuated in the mechanical legs of the robotic exoskeleton. In the process of the data collection, the motor of the actuation system is removed from the integrated actuation system and all the damping components embedded in the joints are also removed to make sure that the user can move freely wearing the robotic exoskeleton. In the stage of data collection, the exoskeleton system is not controlled actively. All collected data are processed by low pass filters before they are saved.

### 2.3. Feature Extraction and Dimension Reduction

#### 2.3.1. Feature Extraction

Since the GRF is close to zero in the swing phase, the collected signals include the GRF in the stance phase and the inclination angles of the leg segment. The combination signal is denoted as:

(1) $$x=\left[F_{s1},F_{s2},F_{s3},\theta_{Ts},\theta_{As},\theta_{T},\theta_{A}\right]$$

where *F_s_*_1_, *F_s_*_2_ and *F_s_*_1_ are the three GRFs of the stance leg, *θ_Ts_* and *θ_As_* are the inclination angles of the thigh and the foot in the stance leg and *θ_T_* and *θ_A_* are the inclination angles of the other leg. Based on the phase identification, the features of the classifier are composed of GRF signals in the swing phase and the inclination angles of the leg segments. The simple amplitude of signals and its phase sequence are limited to identify locomotion modes. Feature extraction is the process of extracting useful information from the filtered signals. Features can be extracted from a time series of sensor signals. A time series is a series of observations of sensors shown as follows:

(2) $$x_{i}(t),\;i=1,\ldots ,m;\;t=1,\ldots ,n$$

where *m* is the number of observations of sensors and *n* is the length of the data from the windows. Features are extracted over the entire window and output at the end of the window. Windows of 150–250 ms for EMG have been suggested as the optimal tradeoff between the classification accuracy and the delay, while 100–250 ms windows can be used for mechanical sensors [27]. There are several feature extraction methods for a time series observations of sensor signals including time-domain, frequency-domain and time-frequency domain [28]. Time-domain features are mainly descriptive statistics and models features. Frequency-domain features are usually derived by a fast Fourier transform (FFT), which cannot deal with non-stationary signals. Time-frequency domain features can obtain features in time and frequency domain at the same time. The most widely used approach is the WPA, in which features are obtained by calculating from the approximation or detail coefficients at specific decomposition levels.

In this paper, a three-layer WPA is adopted to extract features from a time series of observations including signals collected from the pressure sensors and the AHRS sensors due to the fact that wavelet analysis can describe the signal properties in time-frequency domain. The method is a generalization of wavelet decomposition that offers a richer range of possibilities for signal analysis. In wavelet analysis, a signal is split into an approximation and a detail part. Then the approximation part is again split into a second-level approximation and another detail part, so does the detail part, which is repeated to produce more branches like a tree. Wavelet transform (WT) can be used to decompose a signal into sub-bands with low frequency (approximate components) and sub-bands with high frequency (detail components) [29]. The structure of WPD is similar to WT. Both of them have the framework of multi-resolution analysis (MRA) [30]. The main difference between the two techniques is that the WPD can simultaneously break up approximation and detail versions, while WT only breaks up as an approximation version [31]. Based on this theory, the wavelet generating function *δ*(*t*) is determined by the scaling function ϕ(t), which should meet the double-scaling equations [32]:

(3) $$\phi (t)=\sqrt{2}\sum_{k\in N}h_{k}\phi (2t-k),\;\delta (t)=\sqrt{2}\sum_{k\in N}g_{k}\phi (2t-k)$$

where *h_k_* and *g_k_* are the quadrature mirror filters associated with the predefined scaling function and the mother wavelet function respectively. The double scaling equations are only necessary but insufficient conditions; therefore, more constraints should be added to determine *h_k_* and *g_k_* of different wavelets. Define the function set $\left\{v_{m}(t)|m\in N\right\}$ as follows:

(4) $$v_{m}(t)=\left\{ \begin{array}{l} \sqrt{2}\sum_{k\in N}h_{k}v_{m/2}(2t-k),\;if\;m\;is\;even\\ \sqrt{2}\sum_{k\in N}g_{k}v_{(m-1)/2}(2t-k),\;if\;m\;is\;odd \end{array}\right.$$

and:

(5) $$v_{0}(t)=\phi (t),\;v_{1}(t)=\delta (t)$$

The wavelet packet coefficients are given by:

(6) $$C_{p}^{n,m}=2^{n/2}\int_{-\infty }^{+\infty }x(t)v_{m}(2^{n}t-p)dt$$

where *x*(*t*) is the signal, and *n*, *p* and *m* represent the scale, position and surge parameter respectively.

In actual application, the resolution ability of high frequency band signal is expected to be improved. However, the wavelet transform cannot meet the resolution requirement, so WPA is introduced to solve this problem [33]:

(7) $$x(t)=C_{i,0}+C_{i,1}+\cdots +C_{i,j}$$

**Figure 7:**
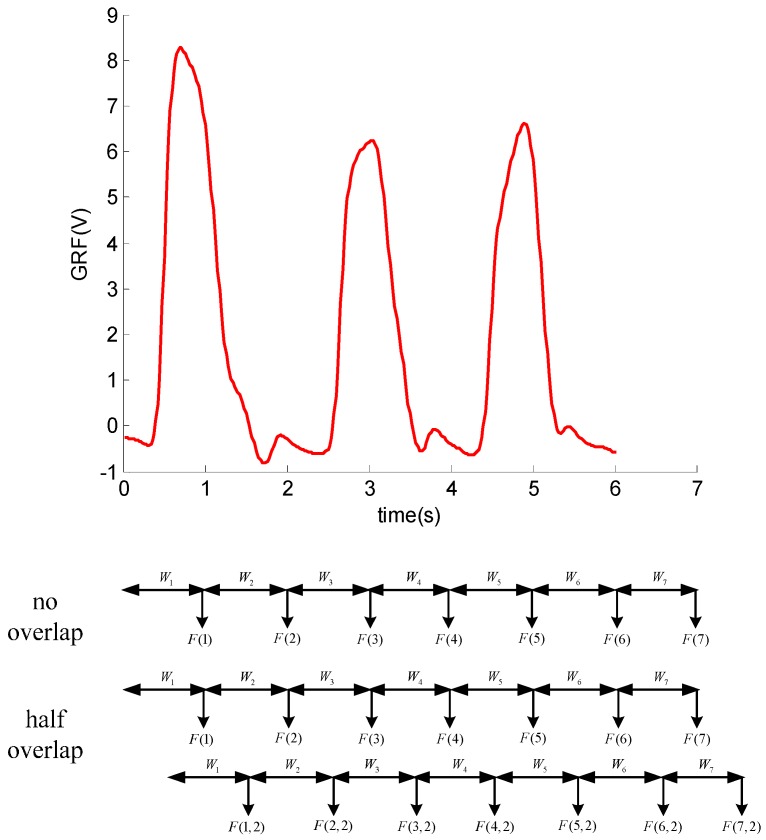
Windowing and feature extraction for a time series of observations. *W*(*T*) is the window of the *T* interval and *F*(*T*) is the corresponding output features. In the case of half overlap, there is a feature set output at each half window, *F*(*T,*2) is the features produced due to overlap.

**Figure 8:**
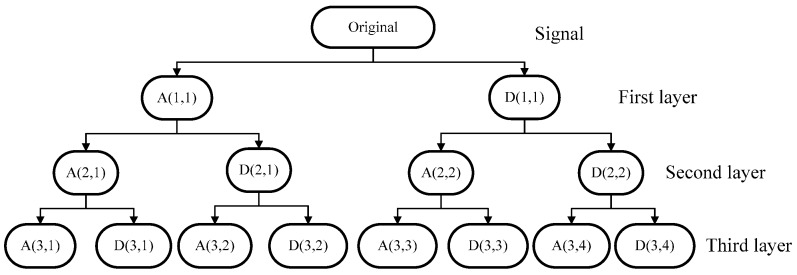
Three layers wavelet packet analysis. *A*(*i,j*) represents the *j*-th approximation coefficient in the *i*-th layer. *D*(*i,j*) is the *j*-th detail coefficient in the *i*-th layer.

As discussed above, the windowing and the features extraction for a time series observations are shown in Figure 7. This decomposition tree of the wavelet packet is shown in Figure 8. 

**Figure 9:**
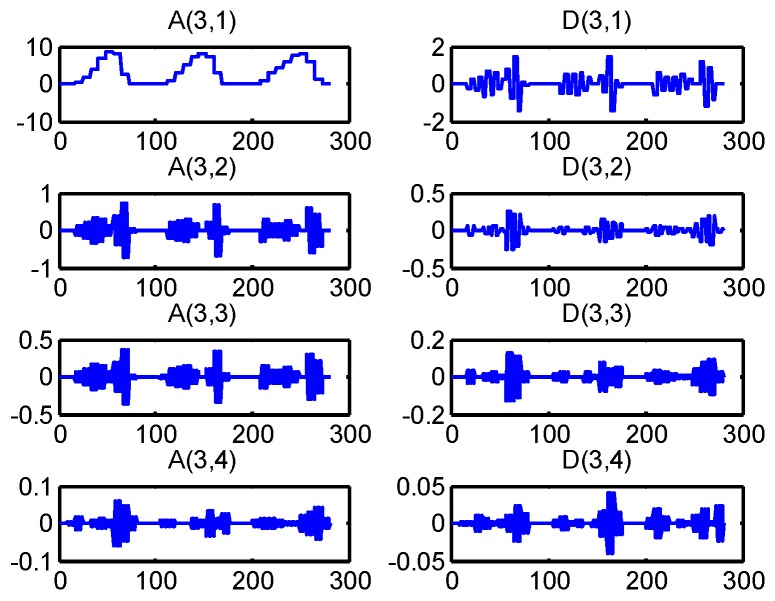
Coefficients of a time series of GRF measured from the heel. The *x*-axis denotes the number of the length of data while the *y*-axis denotes the amplitude of those coefficients.

As depicted in Figure 9, each parent node is divided into two orthogonal subspaces. The features set can be obtained by the three-layer WPA. Those features can show the original signal completely. The three layers wavelet analysis will obtain 14 coefficients. Figure 8 shows the eight coefficients produced by three layers wavelet packet. The wavelet packet decomposition can generate more signal details. For a further analysis of the signal, the energy value of detail signal of wavelet packet decomposition is presented as:

(8) $$E_{i,j}=\int {\left|C_{i,j}\right|}^{2}dt$$

where *E_i,j_* denotes the energy of the decomposition coefficients *C_i,j_*. The feature vectors set can be constructed based on the energy. The feature set can be represented as follows:

(9) $$F_{ea}=\left[E_{i,0},E_{i,1},\cdots ,E_{i,j}\right]$$

To analyze the energy conveniently, the mean energy is used to normalize the feature set in Equation (9), which is depicted as follows:

(10) $$E_{mean}=\sum E_{i,j},F_{ea}^{\prime }=\left[E_{i,0}/E_{mean},E_{i,1}/E_{mean},\cdots ,E_{i,j}/E_{mean}\right]$$

where *E_mean_* is the mean energy of those features and $F_{ea}^{\prime }$ is the new feature set. In this paper, three pressure sensors and four AHRSs are used for the locomotion mode identification. Then the extracted features using WPA for a time series of observations from one single sensor are shown as the following:

(11) $$F={\left(\left\|A(2,1)\right\|,\left\|A(3,2)\right\|,\left\|D(3,2)\right\|,\left\|A(2,2)\right\|,\left\|A(3,4)\right\|,\left\|D(3,4)\right\|\right)}^{T}$$

where ‖·‖ is the norm.

#### 2.3.2. Feature Set Composition

In the classification process using the algorithms such as SVM, it will take a long time to provide an accurate classification when the number of training samples is substantial. Meanwhile, the classification accuracy significantly decreases as the number of features increases [34]. The problems discussed above are related to the curse of dimensionality [35]. As discussed previously, there are six features for each sensor and totally there are 42 features for the feature set. The use of techniques for feature selection, which constructs a smaller features set, will tackle this issue and provide support for accurate classification algorithms. PCA is known as one of the methods that can be used for feature selection, where PCA tries to maximize the variance of original feature vector and aligns the feature vector according to eigenvalues of variance matrix. However, PCA performs a linear separation of data in the original space [36]. Kernel PCA uses the same basic idea as the PCA.

It seeks to project the original features set into a high dimensional space to achieve a nonlinear mapping. Assuming ***F***∈*R^n^* is the feature vector of a time series ***x****_i_*(*t*). Let ***Π*** be the covariance matrix of ***F***, then:

(12) $$\Pi =A\Omega A^{T}$$

where ***Ω*** ***=*** *diag(*[*λ*_1_, …,*λ*_n_]), *λ*_1_ ≥ *λ*_2_ ≥…≥ *λ_n_*, are the eigenvalues of ***Π***, ***A*** is a matrix whose columns are the orthonormal eigenvectors of ***Π***, ***AA****^T^* = ***I****_n_*. General detailed computation process of PCA can be shown in [37]. Let **Φ** be a nonlinear mapping from the original space ***F*** to the final feature space ***F****_s_*, namely **Φ**: ***F***→***F****_s_*. Assuming the kernel matrix ***K*** is a symmetric and positive semi-definite matrix according to Mercer theorem, with the inner product of Φ(*x_i_*) and Φ(*x_j_*) in original space:

(13) $$K_{i,j}=(\Phi (x_{i})\cdot \Phi (x_{j}))=k(x_{i},x_{j}),\;i,j=1,\ldots ,n.$$

where the kernel function is chosen as Gaussian radial basis function (RBF). Let $\bar{\Phi }$ be the center of the final feature space, then:

(14) $${\left(\tilde{K}\right)}_{ij}=((\Phi (x_{i})-\bar{\Phi })\cdot (\Phi (x_{j})-\bar{\Phi }))$$

Combined with (13), $\tilde{K}A=\Omega A$. Then, the following steps of kPCA are similar to those of the ordinary PCA. Before we apply kPCA, the data in ***F*** must be centered. This centering can be conducted as the following:

(15) $$x_{i,\eta }\to x_{i,\eta }-\sum_{\eta }x_{i,\eta }/S$$

where *i* and *η* represent residues and the state labels respectively, and *S* is the total number of the data points. Similar to linear PCA, the ability of reducing the dimensionality of the features set depends on the first few principal components dominating the variance. The direction along the maximum variance can be approximated well with dominant principal components. 

## 3. Locomotion Mode Identification Using SVM Optimized by PSO

### 3.1. SVM for Classifier

**Figure 10:**
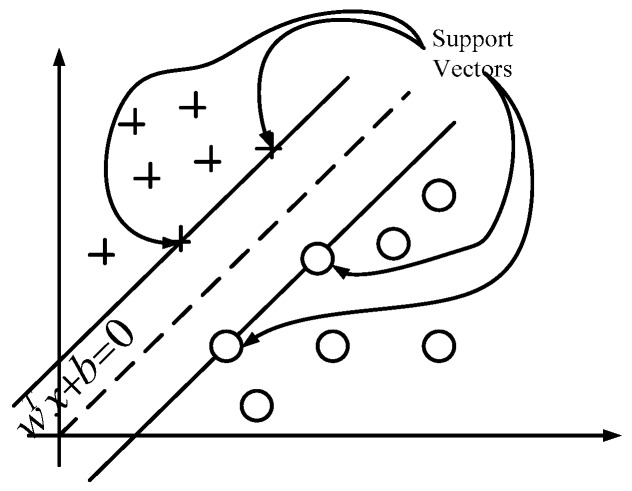
A two-class classification problem by SVM. Data shown by “cross” and “circle” represent binary class +1 and −1, respectively. *w* and *b* are parameters of the hyper-plane.

Support vector machine (SVM) is a general supervised learning method, which is used to solve problems of classification and regression [20]. SVM is considered as a highly efficient classifier and can replace the multi-layer perceptron, RBF neural network, the polynomial network and other existing learning algorithms [38]. The main paradigm for classification problem is to find an optimal separating hyper-plane as a decision boundary, with which the margin of separation among classes can be maximized. As Figure 10 shows, two classes classification problem by SVM is presented. There are two classes labeled +1 and–1, which are represented by “cross” and “circle” respectively. The target of SVM is to find the support vector to separate those two classes. The binary classifier can be extended for multi-classes classifier. For a k-class problem, these methods design a single objective function for training all k-binary SVMs simultaneously and maximize the margins from each class to the remaining ones.

The optimization problem solved by SVM can be presented as follows [37]:

(16) $$\underset{w,b}{\mathrm{minimize}}\frac{1}{2}w^{T}w+C\sum_{i=1}^{N}\max(0,1-y_{i}(w^{T}\Phi (x_{i})+b))$$

where *w* and *b* are the parameters of the hyper-plane. This formulation expressed by Equation (16) can be transformed into the following type:

(17) $$\begin{array}{l} \mathrm{minimize}\;\frac{1}{2}{\left\|w\right\|}^{2}+C\sum \left\|\xi_{i}+\xi_{i}^{*}\right\|\\ \mathrm{subject}\;\mathrm{to}\;\left\{ \begin{array}{l} y_{i}-\langle w,\Phi (x_{i})\rangle -b\leq \varepsilon +\xi_{i}\\ \langle w,\Phi (x_{i})\rangle +b-y_{i}\leq \varepsilon +\xi_{i}^{*}\\ \xi_{i},\xi_{i}^{*}\geq 0 \end{array}\right. \end{array}$$

where *C* is a constant, 〈*w*, *x_i_*〉 denotes the dot product, (*x_i_, y_i_*) is the pair for input and output, $\xi_{i},\;\xi_{i}^{*}$ are slack variables, and Φ is the kernel function, which has multiple forms such as linear kernel, Gaussian RBF kernel, multi-layer perceptron kernel and spline kernel. We can solve Equation (17) by using KKT optimality conditions namely the dual Lagrange method [39]:

(18) $$\begin{array}{l} \mathrm{minimize}\;-\frac{1}{2}\sum_{i=1}^{N}\sum_{j=1}^{N}y_{i}y_{j}a_{i}a_{j}\Phi (x_{i})\Phi (x_{j})+\sum_{i=1}^{N}a_{i}\\ \mathrm{subject}\;\mathrm{to}\;\sum_{i=1}^{N}y_{i}a_{i}=0,\;0\leq a_{i}\leq C,\;\forall i \end{array}$$

where *a_i_* is Lagrange multiplier, and *N* is the number of input features. Let a* be the minimization of this dual problem and suppose the hyper-plane of SVM as *H*(w*,b*). Define [40]

(19) $$I_{S}:=\{i:a_{i}^{*}>0\},\;S:=\{x_{i}:\;i\in I_{s}\}$$

The vectors from *S* are called support vectors and the parameters w* and b* of the hyper-plane are shown as follows:

(20) $$\left\{ \begin{array}{l} w^{*}=\sum_{i\in I_{s}}a_{i}^{*}y_{i}x_{i}\\ b^{*}=y_{i}-\sum_{i\in I_{s}}a_{i}^{*}y_{i}\Phi (x_{i})\Phi (x) \end{array}\right.$$

Then the hyper-plane is found based on Equation (20). The Φ(*x*) is defined as the Gaussian RBF kernel function as follows:

(21) $$\Phi (x)=k(x_{i},x_{j})=\exp(-\sigma {\left\|x_{i}-x_{j}\right\|}^{2})$$

where *σ* is the parameter determining the kernel’s behavior. With the dataset collected, the classifier can be trained to find optimal w* and b*. The hyper-parameters of SVM have an important influence on its performance. The hyper-parameter vector can be defined as $\theta ={\left[a_{i}^{*},\sigma \right]}^{T}$. The determination of the hyper-parameter vector has a significant effect on the performance of the SVM. In this work, the original dataset includes the GRF signals measured by the force sensors and the posture information collected by the AHRS. There are five locomotion modes to be identified, i.e., level-walking, walking downstairs, walking upstairs, walking down ramp and walking up ramp.

### 3.2. PSO-Based SVM

The PSO is similar to the genetic algorithm (GA), both of which apply evolution to find the optimal solution with the initial solution, while the PSO does not have the crossover and mutation operator. In general, the PSO converges to the optimal solution more quickly than the GA [41]. The origin of the PSO is from the concept of the predation on bird populations, one of which is called a particle. All particles mapped into the space evolve with each iteration to obtain the maximum fitness value of the objective function, moving towards the optimal solution. The PSO system is initialized with a population of random solutions and searches for the optima by updating generations. In PSO, the potential solutions, called particles, fly through the problem space by following the current optimum particles. Each particle keeps track of its coordinates in the problem space which are associated with the best solution called *p_best_*. Another best value called *l_best_* which is tracked by the particle swarm optimizer, is obtained so far by any particle in the neighbors of the particle. In PSO, the process of velocity updating is shown as follows [26]:

(22) $$v_{id}^{k+1}=w\cdot v_{id}^{k}+c_{1}\cdot \alpha (p_{id}^{k}-x_{id}^{k})+c_{2}\cdot \beta (p_{gd}^{k}-x_{id}^{k}).$$

where $\;v_{id}^{k+1}$ is the *i*-th particle’s velocity of the next moment, *w* is the inertial weight, *c*_1_ and *c*_2_ are the constants which describe the searching ability for local and global optimization separately, $x_{id}^{k}$ represents the *k*-th particle, $\;p_{id}^{k}$ and $p_{gd}^{k}$ are best values currently and among all particle in the population respectively, *α* and *β* are random values between 0 and 1. Based on the updated velocities, each particle changes its position according to the following equation:

(23) $$x_{id}^{k+1}=x_{id}^{k}+\gamma v_{id}^{k+1}.$$

where *γ* is a constant coefficient.

**Figure 11:**
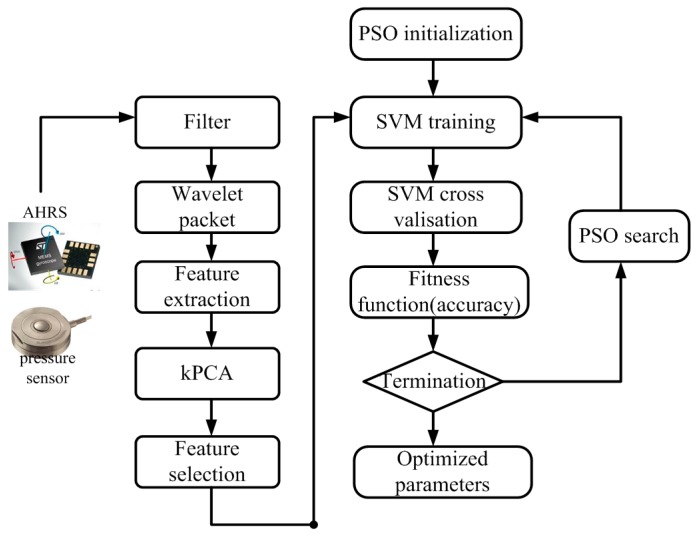
The process of SVM optimization using PSO.

For multi class classification problems, the SVM parameters with soft margins will have significant effects on its accuracy, especially on the penalty constant *C* and the width of the kernel function Φ. Based on the previous discussion, the process of SVM using PSO is illustrated in Figure 11. 

As Figure 11 shows, the determination coefficient, namely the fitness function represented by accuracy of SVM, is a statistical measurement of how well a regression curve approximates real data points. The locomotion mode identification using SVM is relevant to the walking phases, which are segmented into four phase events, i.e., heel strike, stance, heel off and swing. Since the GRF is invalid during the swing phase, the locomotion mode identification is valid only in the phases of heel strike, stance and heel off. 

The PSO-SVM process can be stated as the following steps: (1) Processing the time series of original data to remove noises with a low-pass filter. The collection and the preprocessing are conducted on the central PC; (2) Extracting features from the given data set by WPA. The time series include the GRF signals in the stance phase and the posture information of the double legs; (3) Selecting an optimal feature set with the overall contribution rate of 95%by kPCA to reduce the dimensionality; (4) Labeling the corresponding features set and training the PSO-SVM with 5-fold cross validation to find the optimal hypermeters vector; (5) Obtaining the final trained SVM with a higher testing accuracy; (6) Predicting the locomotion mode label according to the trained SVM online. During the PSO, the fitness function is the training accuracy of SVM and initial generation is set as 10, the maximum generation is 200, c_1_ = c_2_ = 2, and the parameters to be optimized are located in the intervals [0.1, 250] and [0.1, 1200].

### 3.3. Post-Processing Using Majority Voting Algorithm (MVA)

To make sure the classification results are stable, the majority vote algorithm (MVA) is used, which tries to determine whether there is a choice with more occurrences than others in any given sequence of choices, and if so, determine this choice. Supposing the given classification labels sequence is *L*(*n*) = (*l*_1_,*l*_2_,…,*ln*), where *l_i_* is a label representing a locomotion mode. The object of the algorithm is to find the majority number defined as the number that appears most times. The majority voting algorithm is widely used in classification problems to make sure that the classification labels keep consistent during a sequence of fixed size to improve the accuracy of classifier. In this paper, five locomotion modes, i.e., level ground walking, stair ascent, stair descent, ramp ascent and ramp descent, need to be identified, which are labeled as 1, 2, 3, 4, and 5, respectively. The sensor data measured by the sensor in real time can be applied in the online algorithm. The predictive label can be obtained online to illustrate the real locomotion mode. The MVA is applied to improve the identification accuracy of the predictive labels.

## 4. Experiment Protocol and Results Analysis

### 4.1. Experiment Protocol

**Figure 12:**
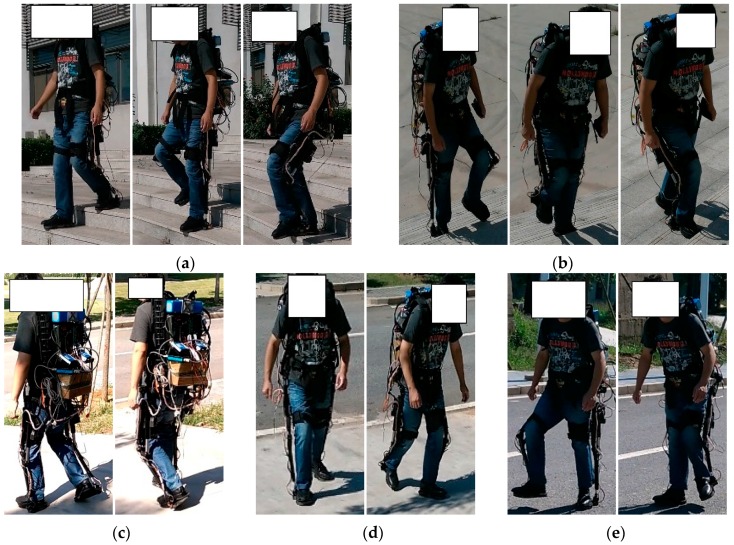
Experiments for locomotion mode identification. (**a**) Stair descent; (**b**) Stair ascent; (**c**) Ramp descent; (**d**) Ramp ascent; (**e**) Level-ground walking.

To evaluate the performance of the proposed method, three healthy (male) subjects participated in the experiment, which is shown in Figure 12. Those three subjects have an average height of 1.70 ± 0.25 m, an average weight of 60 ± 5.6 kg and an average age of 25 ± 1.5 years old. Before the experiments, the sensor system should be checked to make sure all the sensors work well. Then the subjects wear the exoskeleton without actuation and walk on different terrains. The PC will acquire and save the observations of the foot pressure sensors and the AHRS. Before the training dataset was collected, the actuation system on the knee joint was removed from the exoskeleton. Those three subjects were required to walk on the different terrains. Since the transition among those locomotion modes started from the level walking and ended on the floor, e.g., in the case of stair ascent, the wearer walked on the floor initially, performed stairs walking and stopped on the floor finally. 

Each human subject performed two kinds of experiments. The first kind of experiment was to walk in different locomotion modes, i.e., stair descent, stair ascent, ramp descent, ramp ascent and level-ground walking. Each locomotion mode included 10 experimental trials for one human subject. The second kind of experiment is that each experimenter walked from the level ground walking to the other locomotion modes, or vice versa. The second kind of experiment includes eight transitions, of which each transition included 10 experimental trials. 

**Figure 13:**
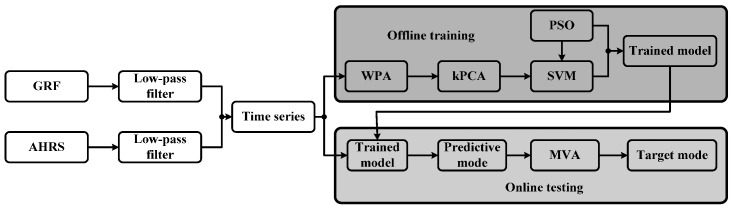
Block diagram of the proposed method. GRF signals and AHRS signals are used to obtain the elements of the time series. The GRFs signals in the swing phase are ignored since it does not change. The trained SVM is obtained offline in MATLAB and the online testing is conducted in Visual Studio.

The process of the proposed PSO-SVM method is shown in Figure 13. Once the training dataset was obtained, the model of PSO-SVM can be trained offline. We separately label several gait patterns, i.e., stair descent, stair ascent, ramp descent, ramp ascent and level-ground as 1, 2, 3, 4 and 5. The stair is 300 cm in width, 36.4 cm in depth, and 15 cm in height while the inclination angle of the 3.5 m long ramp is about 12°. Locomotion mode identification was performed online in real time on the embedded PC and the identified mode can be transferred to the individual PC through the wireless.

### 4.2. Performance Evaluation

Two kinds of indexes, i.e., average identification accuracy (AIA) and identification delay (ID), are used to make reasonable evaluations of identification performance. The first performance index, AIA, is used to evaluate the accuracy of the classifier, which is defined as:

(24) $$AIA=\frac{N_{correct}}{N_{total}}$$

where *N_correct_* is the number of correct identification while *N_total_* is the total number of test events. The usage of the confusion matrix is helpful to quantify the error distribution of the identification, which is defined as the following:

(25) $$C=\left(\begin{array}{l} c_{11},\;c_{12},\;c_{13},\;c_{14},\;c_{15}\\ c_{21},\;c_{22},\;c_{23},\;c_{24},\;c_{25}\\ c_{31},\;c_{32},\;c_{33},\;c_{34},\;c_{35}\\ c_{41},\;c_{42},\;c_{43},\;c_{44},\;c_{45}\\ c_{51},\;c_{52},\;c_{53},\;c_{54},\;c_{55} \end{array}\right)$$

where each element of the confusion matrix is defined as:

(26) $$c_{ij}=\frac{m_{ij}}{{\bar{m}}_{i}}\;\times \;100\%$$

where *m_ij_* is the number of events in the *i*-th locomotion mode that is classified as *j*-th locomotion mode and ${\bar{m}}_{i}$ is the total numberof events in the *i*-th locomotion mode. It is notable that the diagonal elements of the confusion matrix *C* are the identification accuracy and the off-diagonal elements denote the errors. To evaluate whether the transition can be identified in time, the critical moment is defined as the moment when one foot is located in stance phase and the other foot strikes the floor. The ID is defined as:

(27) $$ID=\frac{T_{i}-T_{c}}{T_{gait}}\times 100\%$$

where, *T_i_* is the moment when the locomotion mode transition is identified correctly for the first time, *T_c_* is the critical moment and *T_gait_* is the total time in the walking gait cycle. 

### 4.3. Results Analysis

Here are some abbreviations representing several locomotion modes, e.g., D-S: stair descent; U-S: stair ascent; D-R: ramp descent; U-R: ramp ascent and W: level-ground walking. The three human subjects are required to walk on different terrains at natural speeds. In the control software, the predictive locomotion mode labels are recorded by three different ways, i.e., SVM, PSO-SVM and PSO-SVM with MVA. As discussed previously, the confusion matrix can be used to show the identification accuracy. Three confusion matrices are shown in Table 2, Table 3 and Table 4. 

**Figure 14:**
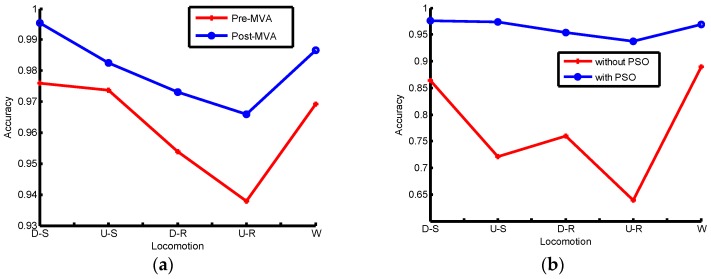
The online identification accuracy comparisons for several locomotion modes: (**a**) comparison between pre-MVA and post-MVA under PSO; (**b**) Comparison between without-PSO and with-PSO under MVA.

**Figure 15:**
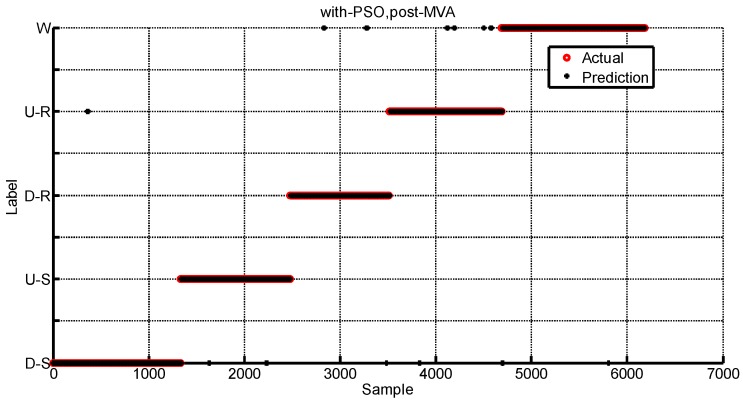
The classification result using PSO-SVM with MVA. Those five instances along the sample axis are those five locomotion modes. Misidentification happens when the actual instances (the circle point) misalign with the prediction instances (the star point).

The elements of confusion matrices are presented by the format of mean and standard error. As Table 1 shows, the maximum identification accuracy happens in the mode of level ground walking at 88.96% and the minimum identification accuracy is about 63.97% during the upstairs walking. Table 2 shows the identification accuracy of the case of PSO-SVM. As Table 2 shows, all locomotion modes have the identification accuracy over 93.8%. The locomotion mode of walking downstairs has the maximum identification accuracy at 97.6%. Compared with Table 1, it is obvious that PSO-SVM has a higher accuracy of the identification. Table 3 shows the identification results by using MVA for the PSO-SVM. The minimum identification accuracy is 97.3% and the maximum is 99.5%. It can be concluded that the application of MVA is effective to improve the identification accuracy. The average identification accuracy can be shown in Figure 14. Figure 14a shows that the post-processing for the identification labels can improve the accuracy and Figure 14b depicts that the optimized SVM is superior. The proposed PSO-SVM with MVA has high identification accuracy. Figure 15 shows the identification result of one human subject using the proposed method. Five locomotion modes can be identified well except several misidentifications. Some instances of D-S are misidentified as U-R, and some instances of U-S are misidentified as D-S, and some instances of D-R are misidentified as D-S or W, and some instances of U-R are misidentified as D-S or W, and some instances of W are misidentified as D-S. 

The performance index of ID is used to evaluate the real-time capability of the proposed identification strategy. In this paper, the transition between locomotion modes happens from the level-ground walking to others, vice versa. Hence, there are eight kinds of transition for locomotion modes, i.e., W to D-S, D-S to W, W to U-S, U-S to W, W to D-R, D-R to W, W to U-R, and U-R to W. The IDs of those transitions are shown in Table 5. As Equation (27) shows, the ID is determined by the *T_i_*, *T_c_* and *T_gait_*. As Table 5 shows, the negative ID shows the *T_i_* is smaller than the *T_c_*, while the positive ID shows the *T_i_* is larger than the *T_c_*. The transition of W to D-S or D-S to W has the largest ID, while the transition of W to U-R, U-R to W has the smallest ID. If the ID is large, the corresponding transition is easy to be misidentified.

## 5. Discussion

An online SVM algorithm with PSO was proposed to identify different locomotion modes in real time for a robotic rehabilitation exoskeleton. In the proposed algorithm, a majority vote algorithm was applied to improve the identification accuracy. Experiments were performed on three human subjects and the experimental results validated that the proposed method is effective for locomotion mode identification.

The proposed method needs six GRF sensors in the wearable shoes and two AHRS sensors on the mechanical parts and there is no need to mount any sensors on the human body, which is very convenient in actual applications. Some work in the locomotion mode identification cares about the steady identification accuracy [12]. This work presents both of steady locomotion mode identification and the transition between them as [18,19] do. In [18], six locomotion modes are identified and the identification accuracy is 99.71% ± 0.05%. Four pressure sensors and three IMUs are placed in the insole and one the leg segment, respectively. However, the experiments are performed on the human subject without any mechanical structure. The accuracy of locomotion mode identification in [19] is 98.74% ± 0.32% and the average identification delay is 9.06% ± 3.46%. The proposed method could achieve accuracy of 98.35% ± 1.65% for different locomotion modes, which is nearly close to the identification accuracy in [19]. The proposed method used WPA to generate a feature set and kPCA to reduce the dimension of the features. Those sensors are placed on mechanical links to reduce the subject fatigue, which is appropriate for long time use. The analysis of identification delay shows that the proposed method can correctly identify all the studied eight transitions between five locomotion modes and the average identification delay is 19.25% ± 2.44% of one gait cycle, which is far less than one step. In general, the transitions between those locomotion modes can be obtained in advance and can control the robotic exoskeleton system to react immediately. 

The proposed method should be improved to solve some problems. The identification delay of the transitions between level ground and stair descent and between level ground and ramp descent is over 30%, which is relatively long and may cause lagging adjustment of the rehabilitation exoskeleton control. The robotic exoskeleton did not consider the speed variation and the transitions between stair descent and stair ascent. In future, the method of identifying the locomotion modes may be applied and extended in the real robotic exoskeleton.

## 6. Conclusions

In this paper, we have proposed to use an online optimal SVM algorithm with PSO to identify five kinds of locomotion modes. With the GRF signals and the AHRS signals, the WPA was used to extract features from the combination time series signals. The experiments were performed on three healthy subjects in the laboratory and the results verified the proposed approach. Two indexes, i.e., identification accuracy and identification delay, were used to illustrate the performance of the proposed method. This paper proposed to combine the GRFs measured by the force sensors in the wearable shoes and the postures of the foot and the shank to be the signal sources for locomotion mode identification. Only two kinds of sensors were applied. The SVM with PSO can improve the identification accuracy by about 20% compared with that only using SVM. Meanwhile, the improvement of accuracy with MVA is nearly 3%. 

## Figures and Tables

**Table 1:** Parameters of the mechanical prototype.

Property-Part	Thigh	Shank
Mass (kg)	0.82	0.6
Length (mm)	431	390
Range of DoF (°)	−37~70	0~75

**Table 2:** Confusion matrix for experimental results of SVM without PSO.

	D-S	U-S	D-R	U-R	W
**D-S**	86.4% ± 1.2%	4.73% ± 0.6%	0.15% ± 0.04%	1.5% ± 0.13%	7.22% ± 1.1%
**U-S**	25.3% ± 0.85%	72.1% ± 1.56%	0.0% ± 0.0%	0.0% ± 0.0%	2.62% ± 0.2%
**D-R**	10.98% ± 1.1%	3.28% ± 0.2%	75.9% ± 1.24%	4.34% ± 0.03%	5.5% ± 0.23%
**U-R**	17.72% ± 1.05%	5.54% ± 0.04%	0.94% ± 0.25%	63.97% ± 2.56%	11.84% ± 0.42%
**W**	7.3% ± 0.25%	2.07% ± 0.13%	0.07% ± 0.01%	1.61% ± 0.13%	88.96% ± 2.1%

**Table 3:** Confusion matrix for experimental results of PSO-SVM.

	D-S	U-S	D-R	U-R	W
**D-S**	97.6% ± 0.85%	1.05% ± 0.05%	0.15% ± 0.03%	0.06% ± 0.0%	0.06% ± 0.01%
**U-S**	2.45% ± 0.1%	97.4% ± 0.56%	0.0% ± 0.0%	0.0% ± 0.0%	0.18% ± 0.06%
**D-R**	1.64% ± 0.22%	0.67% ± 0.1%	95.4% ± 1.14%	0.39% ± 0.01%	1.93% ± 0.15%
**U-R**	1.87% ± 0.03%	0.6% ± 0.0%	0.26% ± 0.02%	93.8% ± 0.25%	3.5% ± 0.24%
**W**	1.94% ± 0.11%	0.6% ± 0.02%	0% ± 0.0%	0.54% ± 0.02%	96.9% ± 0.86%

**Table 4:** Confusion matrix for experimental results with MVA under PSO.

	D-S	U-S	D-R	U-R	W
**D-S**	99.5% ± 0.05%	0.0% ± 0.0%	0.0% ± 0.0%	0.05% ± 0.05%	0.0% ± 0.0%
**U-S**	0.34% ± 0.01%	98.3% ± 0.62%	0.0% ± 0.0%	0.85% ± 0.05%	0.51% ± 0.02%
**D-R**	0.01% ± 0.01%	0.58% ± 0.08%	97.3% ± 0.45%	0.0% ± 0.0%	2.02% ± 0.05%
**U-R**	0.77% ± 0.1%	0.0% ± 0.0%	0.51% ± 0.12%	97.36% ± 0.66%	2.13% ± 0.13%
**W**	0% ± 0%	0.0% ± 0.0%	0.0% ± 0.0%	0.47% ± 0.11%	98.66% ± 0.24%

**Table 5:** DIs of locomotion mode transitions with the proposed strategy.

Transition	ID
W to D-S	48% ± 2.8%
D-S to W	46.8% ± 4.5%
W to U-S	−10.4% ± 1.2%
U-S to W	−6.4% ± 0.8%
W to D-R	31.5% ± 2.45%
D-R to W	40.5% ± 5.2%
W to U-R	2.5% ± 0.8%
U-R to W	4.5% ± 1.8%
